# MRI-guided neurolysis for the treatment of chronic refractory knee pain: a case report

**DOI:** 10.1007/s00256-024-04819-9

**Published:** 2024-11-12

**Authors:** Alexander W. Marka, Maximillian Strenzke, Kilian Weiss, Dimitrios C. Karampinos, Klaus Woertler, Mirco Herbort, Nima Befrui, Tom Finck

**Affiliations:** 1 Present Address: Department of Diagnostic and Interventional Radiology, School of Medicine & Klinikum rechts der Isar, Technical University of Munich, Ismaninger Str. 22, 81675 Munich, Germany; 2 Musculoskeletal Radiology Section, School of Medicine & Klinikum Rechts Der Isar, Technical University of Munich, Ismaninger Str. 22, 81675 Munich, Germany; 3 Philips GmbH, Röntgenstrasse 22, 22335 Hamburg, Germany; 4 Orthopädische Chirurgie München – OCM, Steinerstraße 6, 81369 Munich, Germany

**Keywords:** MRI, Knee, Chronic knee pain, Neurolysis

## Abstract

Chronic refractory pain poses a significant challenge in knee joint pathologies, especially after exhaustion of conservative, arthroscopic, and endoprosthetic therapy options. This case report illustrates an innovative approach using MRI-assisted chemical neurolysis of a genicular nerve to manage persistent knee pain after arthroscopy. A 62-year-old male patient with chronic refractory knee pain, primarily localized at the inferomedial part of the knee, underwent high-resolution MRI to visualize the genicular nerves. This allowed for targeted ethanol-based neurolysis of the inferomedial genicular nerve. Following the procedure, the patient experienced substantial pain reduction for the follow-up duration of 4 months. The successful use of MRI-assisted chemical neurolysis offers a promising alternative treatment for patients with refractory knee pain, providing long-lasting pain relief without major side effects. This technique has the potential to improve the quality of life for patients suffering from chronic knee pain While these initial results are encouraging, it is important to note that further research, including both short-term and long-term studies, as well as randomized controlled trials, is warranted to establish the efficacy and safety of this treatment method in broader populations before it can be considered for routine incorporation into pain management practices.

## Introduction

Chronic knee pain, particularly in patients with osteoarthritis, those who have undergone multiple arthroscopic knee surgeries, or those experiencing persistent pain following total knee arthroplasty, is a common condition that can significantly impair quality of life. It often persists despite various non-invasive (physical therapy, medication) or invasive (arthroscopy, total joint replacement) treatments [1]. One of the major challenges in locally addressing chronic knee pain is the inability to reliably target the periarticular nerves using ultrasound or fluoroscopy, although alternative pain management approaches, including radiofrequency ablation and chemical neurolysis of the genicular nerves, have shown promising results in previous studies [2–4].

**Fig. 1:**
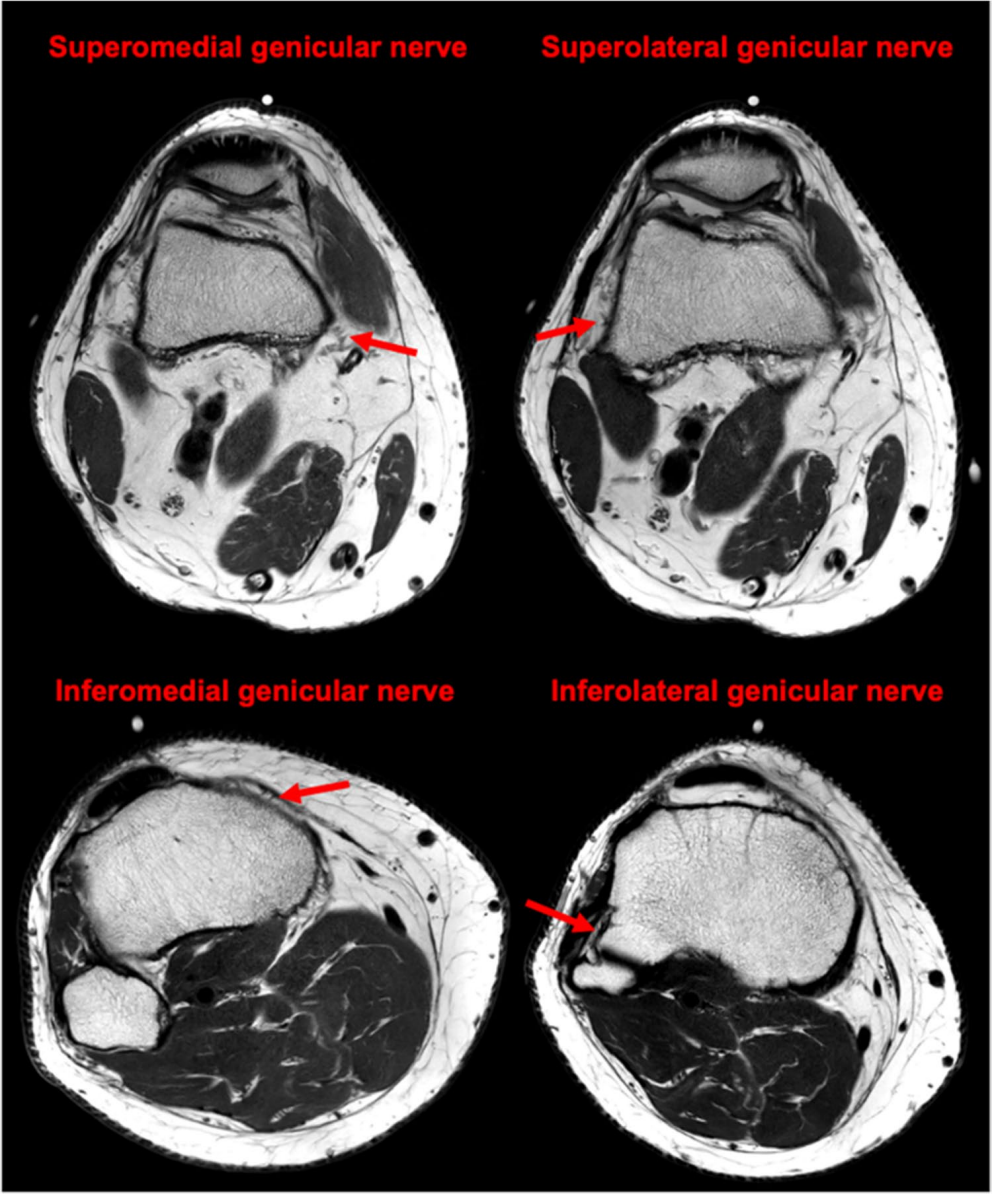
Axial T2-weighted high-resolution MRI shows the superomedial, superolateral, inferomedial, and inferolateral genicular nerves. Imaging details: 5:30 min acquisition, 2.5 mm slice thickness, 0.33 × 0.29 mm resolution, with compressed sensing and deep learning reconstructions

Recent advances in imaging technology, in particular high-resolution MRI, have greatly improved the visualization of complex knee anatomy [5]. This includes the ability to clearly delineate the small genicular nerves, which supply the knee capsule, and which are thought to play a significant role in transmitting pain signals. High-resolution knee MRI provides a detailed view of the genicular nerves, including their branching patterns and proximity to other structures. This may reduce the risk of unwanted intra-articular injection or peroneal nerve ablation and increase the likelihood of successful outcomes with more precise targeting of the nerve. Figure 1 highlights the anatomy of the superomedial, superolateral, inferomedial, and inferolateral genicular nerves in the patient discussed in this case report.

## Case report

A 62-year-old male had been experiencing chronic knee pain for several years, which was attributed to deep cartilage damage on the medial condyle, as shown by MRI and confirmed by arthroscopy. Despite undergoing matrix-associated autologous chondrocyte transplantation (MACT) in July 2023, the patient continued to experience persistent pain in the inferomedial aspect of the knee. The pain (8/10 on a numeric rating scale (NRS) for pain) was exacerbated in knee flexion and accompanied by peripatellar tension. The patient reported significant impairment of daily activities, especially while walking stairs, leading to a reduction of quality of life and limitation of rehabilitation training. After unsuccessful attempts to manage the pain through a multidisciplinary approach, including physiotherapy and infiltration with local anesthesia and corticosteroids, the patient was referred from an outpatient orthopedic clinic for further evaluation and management.

We performed a high-resolution planning MRI (acquisition time: 5:30 min, slice thickness: 2.5 mm, in-plane resolution: 0.33 × 0.29 mm, acceleration technique: compressed sensing with a sense factor of 6 and deep learning-based reconstructions) of the knee to visualize the genicular nerves innervating the knee capsule. This imaging allowed us to precisely locate the nerves, particularly the inferomedial genicular nerve, which was likely contributing to the patient’s pain.

The procedure was conducted using a standard 3 T MRI scanner, with a routine diagnostic MRI slot blocked off for approximately 30 min. No dedicated procedural MRI suite was required. We used MRI-compatible equipment, including non-ferromagnetic needles, to ensure safety within the MRI environment. The procedure involved precise nerve localization using high-resolution MRI, followed by real-time guided administration of the neurolytic agent.

**Fig. 2:**
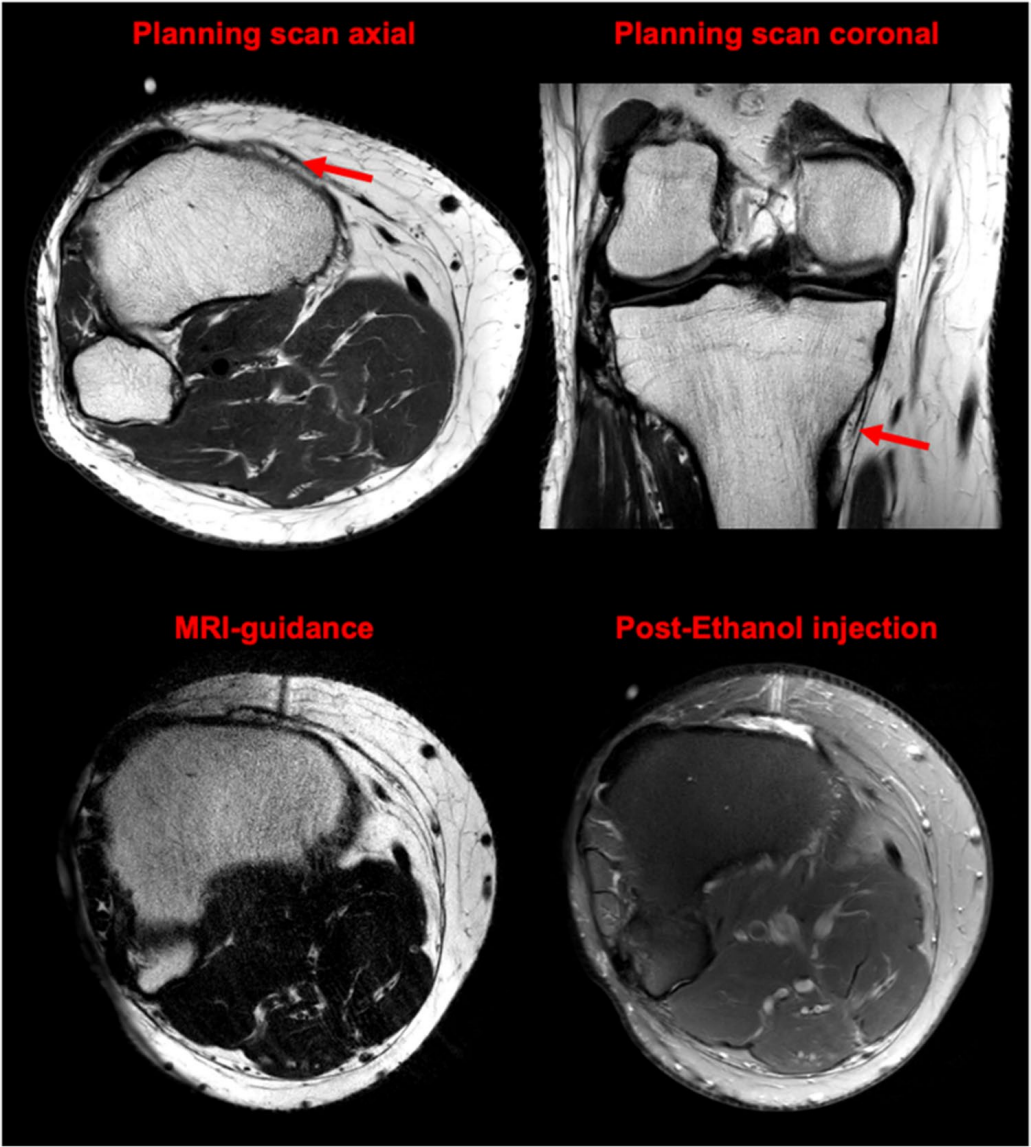
Top: Axial and coronal planning scans showing the inferomedial genicular nerve (red arrows). Bottom left: Axial T2 navigating scans (33 s, 3 mm slice, 0.33 × 0.29 mm resolution) using compressed sensing and deep learning. Bottom right: Axial postprocedural STIR images confirm targeted ethanol deposition around the inferomedial genicular nerve

After obtaining informed consent, the exact location of the inferomedial nerve was targeted for chemical neurolysis. For this, limited-coverage navigating scans were used (acquisition time: 33 s, slice thickness: 3 mm, in-plane resolution: 0.33 × 0.29 mm, acceleration technique: compressed sensing with a sense factor of 3 and deep learning-based reconstructions). Initial injection of 1 ml Bupivacaine 1% confirmed the patient’s pain reduction without any impact on muscular function. Encouraged by this response, the patient returned the following week for a targeted ethanol neurolysis procedure, during which 1 ml of 97% ethanol mixed with 0.5 ml of Bupivacaine was administered. Procedural planning scans, clearly depicting the inferomedial genicular nerve and an MRI needle guidance scan are shown in Fig. 2. Postprocedural STIR images confirmed that ethanol deposition was performed solely around the target nerve and did not extend beyond that area. The procedure was performed under local anesthesia only and conducted in an ambulatory setting, allowing the patient to leave after a brief post-procedure observation period.

After the procedure, the patient reported significant pain reduction (8/10 -> 1/10 on an NRS) which persisted until the end of follow-up after 4 months (1/10 on an NRS). While there was still a feeling of slight peripatellar tension, the knee function, especially the ability to climb stairs, was regained.

## Discussion

Chronic knee pain can be challenging to treat, particularly if conventional therapies fail to provide relief. This case report highlights the potential benefits of using MRI-assisted chemical neurolysis for managing refractory knee pain.

High-resolution MRI allows for clear visualization and subsequent targeting of genicular nerves. This approach maximizes the likelihood of successful pain relief while minimizing the risk of damage to surrounding structures (4). In our case, the ability to identify the inferomedial genicular nerve and its branches enabled targeted chemical neurolysis, which contributed to the significant and lasting pain reduction observed in the patient.

Chemical neurolysis with ethanol is a safe and effective method for managing nerve-related pain. Current approaches for genicular nerve identification during ablation are ultrasound- or fluoroscopy-based, with success rates defined as > 50% improvement of pain intensity at 3 months in up to 60% of patients [4]. However, these methods rely on indirect anatomical landmarks, which can result in uncertainty regarding the precise location of the target nerve, potentially leading to suboptimal outcomes or inadvertent damage to surrounding structures.

In this particular case, high-resolution MRI was selected as the imaging modality over fluoroscopy and ultrasound due to its superior capability in precisely localizing nerves through detailed soft tissue imaging. Unlike fluoroscopy, which depends on visible anatomical landmarks that may not accurately correspond to the nerve’s exact location, and ultrasound, whose effectiveness is highly dependent on the operator’s expertise, MRI offers a more consistent and reliable approach. This precision ensures that the neurolytic agent is delivered exactly where needed, minimizing the risk of affecting surrounding tissues, including the potential for accidental motor blockade. Additionally, the use of MRI allows for radiation-free therapy.

Regarding prior treatment options, the patient had not undergone conventional genicular nerve ablation with radiofrequency or genicular artery embolization. The decision to forego genicular artery embolization was based on the patient’s concerns about the risks associated with this more invasive treatment. Radiofrequency ablation was also not considered, as it is not compatible with MRI. The choice of MRI-guided chemical neurolysis was made with the intention of achieving effective pain relief while minimizing the risks and uncertainties associated with other imaging modalities and treatment options.

Moreover, MRI guidance can facilitate the targeting of nerves that are traditionally more challenging to approach, such as the inferolateral genicular nerve, which is often spared due to its proximity to the common peroneal nerve, and the patellar branch of the saphenous nerve. By providing clearer visualization of these nerves, MRI guidance could expand the range of treatable targets, leading to more comprehensive and effective pain management strategies.

Further studies are needed to explore the long-term outcomes and safety of this treatment in larger populations. While this case report provides initial insights into the potential benefits of MRI-assisted chemical neurolysis, more comprehensive data is required before considering its inclusion in management algorithms for refractory knee pain.
